# Dynamic change in phosphorylated platelet-derived growth factor receptor in peripheral blood leukocytes following docetaxel therapy predicts progression-free and overall survival in prostate cancer

**DOI:** 10.1038/sj.bjc.6604706

**Published:** 2008-10-07

**Authors:** P Mathew, P F Thall, S Wen, C Bucana, D Jones, E Horne, W K Oh, M J Morris, Y-C Lee, C J Logothetis, S-H Lin, I J Fidler

**Affiliations:** 1 Department of Genitourinary Medical Oncology, MD Anderson Cancer Center, 1155 Pressler, Houston, TX 77030, USA; 2 Department of Biostatistics, 1400 Holcombe Blvd, Houston, TX 77030, USA; 3 Department of Cancer Biology, MD Anderson Cancer Center, 1515 Holcombe Blvd, Houston, TX 77030, USA; 4 Lank Center for Genitourinary Oncology, Dana-Farber Cancer Institute, 44 Binney St, Boston, MA 02115, USA; 5 Genitourinary Oncology Service, Department of Medicine, Memorial Sloan Kettering Cancer Center, New York, NY, USA; 6 Department of Molecular Pathology, MD Anderson Cancer Center, 1515 Holcombe Blvd, Houston, TX 77030, USA

**Keywords:** prostate cancer, platelet-derived growth factor receptor, docetaxel, survival

## Abstract

In a placebo-controlled randomised study of the platelet-derived growth factor receptor (PDGFR) inhibitor imatinib mesylate and docetaxel in metastatic prostate cancer with bone metastases (*n*=116), no significant differences in progression-free and overall survival were observed. To evaluate pharmacodynamic correlates of outcomes, we assessed the association of plasma platelet-derived growth factor (PDGF) isoform kinetics and PDGFR inhibition with progression-free and overall survival by individual treatment arm. We found that in the docetaxel–placebo arm *alone*, the probability of decrease in PDGFR phosphorylation (Pr-Decr-pPDGFR) above 0.5 (*vs* ⩽0.5) was associated with a sharp increase in all measured plasma PDGF isoforms (*P*=0.006 for AA, 0.002 for BB, 0.045 for AB); a decreased median progression-free survival of 3.3 months *vs* 6.8 months (hazard ratio (HR) 2.5; *P*=0.006 in log-rank test) and an inferior median overall survival of 20 months *vs* >30 months (HR 3.1; *P*=0.04 in log-rank test). By contrast, in the docetaxel plus imatinib arm, the association of Pr-Decr-pPDGFR >0.5 with a rise in plasma PDGF isoform concentrations and inferior survival was not observed. The data suggest that dynamic changes in PDGFR phosphorylation in peripheral blood leukocytes predict docetaxel efficacy. Rising plasma PDGF concentrations may explain and/or mark docetaxel resistance. Validation and mechanistic studies addressing these unexpected findings should anticipate a confounding influence of concurrent PDGFR inhibitor therapy.

Preclinical (Uehara *et al*, 2003; Kim *et al*, 2006) and early clinical (Mathew *et al*, 2004) studies suggested a benefit for the combination of therapeutic inhibition of the platelet-derived growth factor receptor (PDGFR) and taxane chemotherapy for bone metastases from prostate cancer. In a randomised placebo-controlled clinical trial of PDGFR inhibition with imatinib mesylate (Buchdunger *et al*, 2000) and docetaxel chemotherapy in men with castration-resistant prostate cancer and bone metastases, high-frequency expression of the target – phosphorylated PDGFR in metastatic tumour in bone – was confirmed, with the evidence of enhanced systemic inhibition of PDGFR phosphorylation measured in peripheral blood leukocytes and significant reductions in urine N-telopeptide, a bone lysis marker, in the docetaxel plus imatinib arm compared with docetaxel plus placebo arm. However, there was no clinical benefit, as assessed by progression-free survival (PFS) and overall survival, to support a definitive larger randomised study (Mathew *et al*, 2007).

To evaluate pharmacodynamic correlates of PDGFR inhibition with therapeutic results, we assessed the kinetics of circulating platelet-derived growth factor (PDGF) dimeric isoforms and PDGFR inhibition in peripheral blood leukocytes and their association with PFS and overall survival in the control and imatinib-containing arms. Current knowledge indicates that the platelet-derived growth factors (PDGFs) consist of five different dimeric isoforms (AA, BB, AB, CC, and DD) derived from four different polypeptide chains encoded by four different genes acting via two receptor tyrosine kinases, PDGFR *α* and *β* (Fredriksson *et al*, 2004; Reigstad *et al*, 2005). PDGF functions have been implicated in a wide range of physiological and pathological processes including those relevant to neoplasia such as angiogenesis, inflammation and mesenchymal differentiation (Pietras *et al*, 2003; Yu *et al*, 2003). Although circulating ligand kinetic profiles have not been described in the context of PDGFR inhibition in neoplasia, effective therapeutic inhibition of a range of other receptor tyrosine kinases, including epidermal growth factor receptor or the vascular endothelial growth factor receptor, have been associated with particular variations in circulating ligand concentrations (Burdick *et al*, 2000; Bocci *et al*, 2004; DePrimo *et al*, 2007).

## Materials and methods

### Description of cohort

A total of 116 men with castration-resistant metastatic prostate cancer were accrued from 28 April 2003 through 19 August 2005 at five tertiary cancer care centres as described earlier (Mathew *et al*, 2007). Eligibility criteria included histologic evidence of adenocarcinoma of the prostate with radiological evidence of bone metastases, a serum testosterone level of ⩽50 ng dl^−1^, and evidence of disease progression as manifested by either successive increases in serum prostate-specific antigen (PSA) level, the appearance of new lesions on bone scan, progressive bidimensional disease and/or worsening malignant bone pain. All patients provided written informed consent according to institutional guidelines. The study was supported by Novartis Pharmaceuticals and an Inter-Specialised Program of Research Excellence (SPORE) grant from the National Cancer Institute.

### Therapeutics

Patients were randomly assigned to receive docetaxel at 30 mg m^−2^ administered intravenously over 60 min, on days 1, 8, 15, and 22 in 42-day cycles, along with daily oral imatinib mesylate 600 mg (D+I) or placebo (D+P). Therapy was to continue until disease progression was established. On account of toxicity, the starting dose of imatinib was later lowered to 400 mg daily.

### Samples for correlative studies

Baseline laboratory studies included an optional peripheral blood sample for biomarker monitoring of PDGFR inhibition. Serial peripheral blood samples for monitoring phosphorylated-PDGFR (pPDGFR) were repeated on day 1 of cycle 2 (C2D1) prior to docetaxel administration, in patients who consented to provide these optional research samples. There were 88 paired samples (41 D+I, 47 D+P) for peripheral blood leukocyte PDGFR phosphorlation assay. There were 102 samples at baseline (50 D+I and 52 D+P) for PDGF ligand assay and 89 of these (42 D+I, 47 D+P) had a paired sample available after therapy. Correlations of pPDGFR and plasma PDGF kinetics were feasible among 88 paired samples.

### Measurement of PDGF ligands

An enzyme-linked immunosorbent assay system was used to determine the concentration of plasma PDGF-AA, PDGF-BB, and PDGF-AB (R&D Systems, Minneapolis, MN, USA). All measurements of ligand concentration (ng ml^−1^) were computed averages from duplicate assays in two different enzyme-linked immunosorbent assay plates.

### Assay of pPDGFR expression in peripheral blood leukocytes

**Figure 1:**
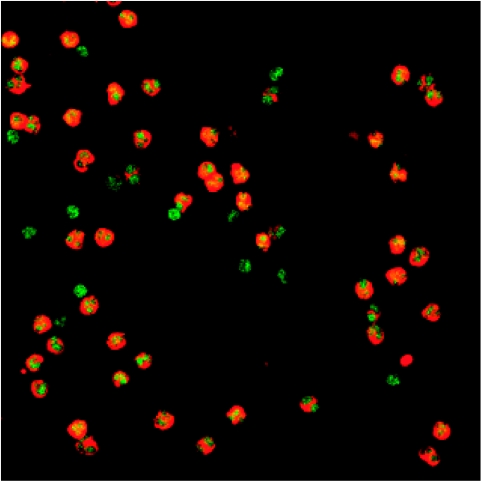
Phospho-PDGFR expression in peripheral blood leukocytes by immunofluorescent antibody staining.

Venous blood samples were drawn at baseline and at C2D1. Cytospin preparations of peripheral blood leukocytes were stained with the pPDGFR-*β* (tyr 1021) antibody (Santa Cruz Biotechnology, Santa Cruz, CA, USA) conjugated to cyanine-5 by Rockland Immunochemical Co. (Gilbertsville, PA, USA) and examined by confocal microscopy (Mathew *et al*, 2007). An example of a baseline specimen from a patient is demonstrated in Figure 1. A laser scanning cytometer (Compucyte Corporation, Cambridge, MA, USA) was used to measure fluorescence intensity of 2000 individual peripheral blood leukocytes and histograms were generated for analysis.

### Statistical analysis

Paired samples of pPDGFR values from peripheral blood leukocytes were available at baseline and on C2D1 from each patient. Using these paired samples, we estimated the probability, Pr(Decr-pPDGFR), that the pPDGFR levels within each patient decreased from baseline to C2D1 and assessed the ability of the estimated Pr(Decr-pPDGFR) values to predict PFS (Mathew *et al*, 2007). The large sample sizes (approximately 2000 cells each) of cell-specific pPDGFR values obtained at both measurement points for each patient provide highly reliable within-patient estimators of Pr(Decr-pPDGFR). Each patient's Pr(Decr-pPDGFR) estimator was based on a Wilcoxon–Mann–Whitney statistic (Wilcoxon and Wolfe, 1979). Descriptive statistical analyses were carried out using histograms, box plots, means, and s.d. Scatter plots, non-parametric regression using lowess smoothers (Cleveland, 1979; Cleveland and Devlin, 1988), linear regression and Pearson's correlation (Neter *et al*, 1990) were used to assess association between numerical variables. Wilcoxon signed-rank tests for paired data (Hollander and Wolfe, 1979) were used to assess ligand changes between baseline and C2D1 within treatment groups. Wilcoxon rank-sum tests (Hollander and Wolfe, 1979) were used to compare unpaired ligand measurements at baseline to those on C2D1. Unadjusted survival probabilities were estimated using the Kaplan–Meier method (Kaplan and Meier, 1958). The two-sided log-rank test (Mantel, 1966) was used to compare PFS between treatment groups. The Cox proportional hazards model (Cox, 1972) was employed to evaluate the prognostic importance of covariates. The Grambsch–Therneau test (Grambsch and Therneau, 1994) was used to assess the proportional hazard assumption for the Cox regression model. All *P*-values were derived from two-sided tests. All computations were performed using SPLUS 2000 (Insightful Corporation, Seattle, WA, USA). To facilitate explanation of the results, specific regression models involving PDGF kinetics are given below, in the Results section.

## Results

### Plasma PDGF dimer distributions

We assessed means and s.d. of PDGF dimer values (AA, AB, and BB) at baseline by treatment group (D+I *vs* D+P). The results (Table 1) show no statistically significant differences between the two groups.

### Plasma PDGF dimer kinetics

**Figure 2:**
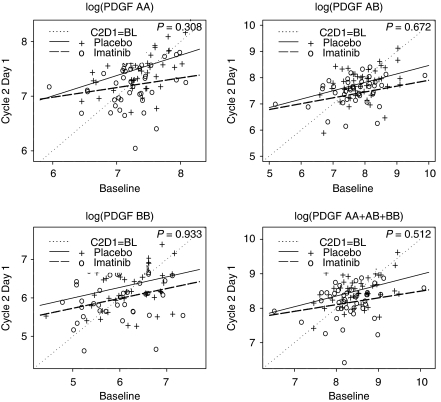
Scatter plots and fitted lines of plasma PDGF kinetics for isoforms AA, AB, BB, and the sum AA+AB+BB, for each treatment arm. The dotted 45° line is a reference corresponding to no differences between the cycle 2 day 1 (C2D1) and baseline (BL) values.

To assess changes in plasma PDGF dimeric isoform concentrations (AA, AB, BB, and total) after one cycle of therapy, denoting the baseline value as X and the value on C2D1 as Y, we fit the linear model Y=*μ*+*α* X+(*β*+*γ* X)^*^[Imat]+*ε*, where [Imat]=1 in the D+I group and 0 in the D+P group; *α*, *β*, and *γ* are parameters, and *ε* is measurement error. Thus, the C2D1 (Y) value is assumed to be a linear function of the baseline (X) value within each treatment group, with average values (*μ*+*β*)+(*α*+*γ*)X in the D+I group and *μ*+*α*X in the D+P group, that is, straight lines having both different slopes and different intercepts. Thus, the imatinib effect is the difference {(*μ*+*β*)+(*α*+ *γ*)X}−{*μ*+*α*X}=*β*+*γ*X, which also is a linear function of the baseline value X. Figure 2 shows scatter plots and fitted models of C2D1 (Y) values with baseline (X) for each PDGF dimer set. Although the C2D1 D+P values were on average higher than the D+I values, no significant differences were noted between the treatment groups, and C2D1 values were on average much smaller than baseline values for each patient.

### Plasma PDGF dimer kinetics and PDGFR phosphorylation status by treatment arm

Because variations in ligand kinetics may be uniquely dependent upon quantitative receptor inhibition, we assessed plasma PDGF dimer kinetics in relation to variations in PDGFR phosphorylation in the control and imatinib-containing arms. The following piecewise linear regression model was fit to each change in dimer value D=Y−X 
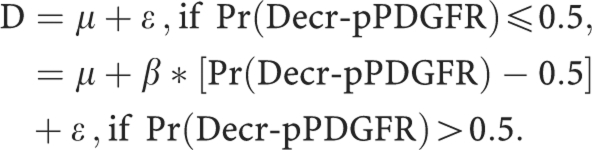


**Figure 3:**
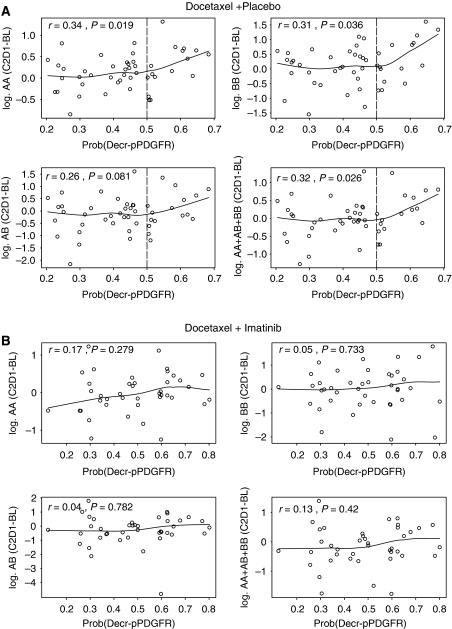
Plasma PDGF ligand kinetics and pPDGFR dynamics in peripheral blood leukocytes by treatment arm: (**A**) Docetaxel and Placebo (**B**) Docetaxel and Imatinib. BL=baseline; C2D1=cycle 2 day 1.

This model was suggested by the non-parametric smoothed plots given in Figure 3A. For the D+P arm, all slopes (*β*) for values of D where Pr(Decr-pPDGFR) >0.5 were negative, and statistically the slopes were significantly different from 0 for each PDGF dimer set (*P*=0.006 for AA, 0.002 for BB, 0.045 for AB, and 0.009 for total ligand). In contrast, for the D+I group (Figure 3B), the slopes (*β*) were not significantly different from 0 (*P*=0.334 for AA, 0.840 for BB, 0.527 for AB, and 0.303 for total ligand).

### PDGFR phosphorylation status and PFS by treatment arm

**Figure 4:**
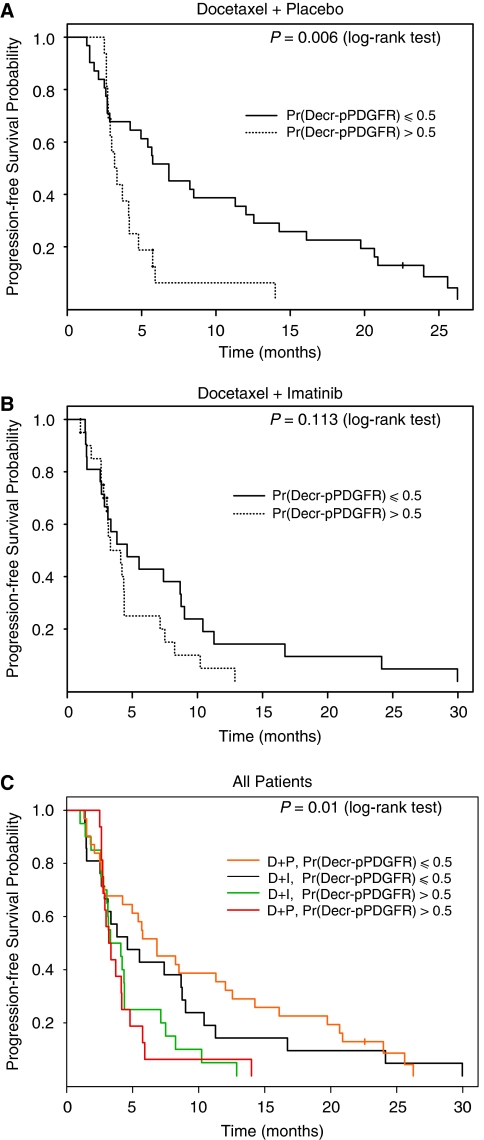
Progression-free survival and pPDGFR dynamics in peripheral blood leukocytes by treatment arm: (**A**) Docetaxel and Placebo (**B**) Docetaxel and Imatinib, and (**C**) all patients. Pr(Decr-pPDGFR)=probability of decrease in phosphorylated PDGFR.

With the same cut point of Pr(Decr-pPDGFR) >0.5, at which rising plasma PDGF concentrations were observed, PFS outcomes were estimated. There was a large and statistically significant difference in PFS between patients with Pr(Decr-pPDGFR) ⩽0.5 and those with Pr(Decr-pPDGFR) >0.5 (hazard ratio (HR) is 2.5 with 95% CI: 1.3–5.1; *P*=0.006 in log-rank test) in the D+P group. The median PFS duration was 6.8 months (95% CI: 4.2–14.3) in patients with Pr(Decr-pPDGFR) ⩽0.5 and was 3.3 months (95% CI: 2.8–5.8) in patients with Pr(Decr-pPDGFR) >0.5 (Figure 4A). In contrast, no statistically significant differences were seen in the D+I group with a median PFS duration of 4.2 months in both patients with Pr(Decr-pPDGFR) ⩽0.5 and those with Pr(Decr-pPDGFR) >0.5 (HR is 1.7 with 95% CI: 0.88–3.3; *P*=0.113 in log-rank test) (Figure 4B). The combined effects of both Pr(Decr-pPDGFR) ⩽0.5 and treatment on PFS, however, are shown by the four Kaplan–Meier curves in Figure 4C, which indicates that the advantageous effect of Pr(Decr-pPDGFR) ⩽0.5 *vs* Pr(Decr-pPDGFR) >0.5 was greater than any treatment effect. In the multivariate analysis, a Cox regression model was fit for PFS including treatment arm assignment, an indicator of Pr(Decr-pPDGFR) >0.5 interacting with each treatment arm, haemoglobin, serum alkaline phosphatase, history of any prior chemotherapy exposure, and baseline Eastern Cooperative Oncology Group (ECOG) Performance Score. The proportional hazard assumption was valid (*P*=0.612). Independent factors predicting PFS (Table 2) were Pr(Decr-pPDGFR) >0.5 (HR=2.419, *P*=0.015 in Placebo group; HR=1.998, *P*=0.037 in Imatinib group), haemoglobin ⩾11 g dl^−1^ (HR=0.300, *P*=0.005), and serum alkaline phosphatase (HR=1.59, *P=*0.061).

### PDGFR phosphorylation status and overall survival by treatment arm

**Figure 5:**
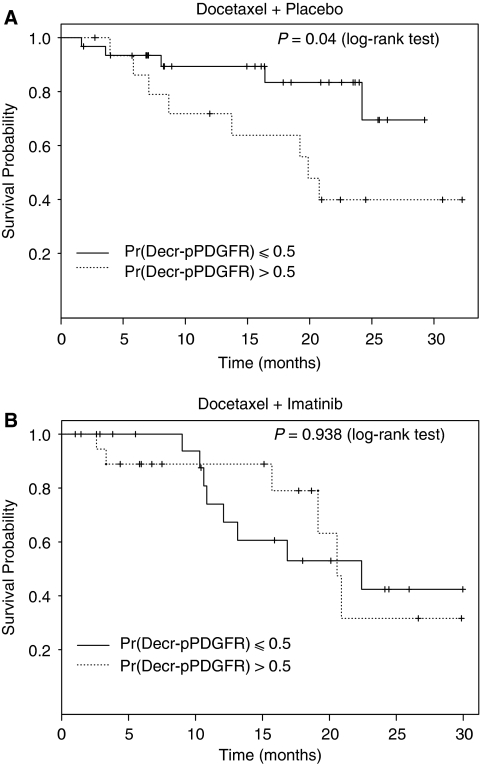
Overall survival and pPDGFR dynamics in peripheral blood leukocytes by treatment arm: (**A**) Docetaxel and Placebo (**B**) Docetaxel and Imatinib. Pr(Decr-pPDGFR)=probability of decrease in phosphorylated PDGFR.

We similarly assessed the association of Pr(Decr-pPDGFR) >0.5. with overall survival outcomes by treatment arm and found that in the D+P group, a Pr(Decr-pPDGFR) >0.5 was associated with a median overall survival of 20 months (95% CI: 13.7 to 30+) *vs* >30 months (median, not reached) when Pr(Decr-pPDGFR) was ⩽0.5 (HR is 2.5 with 95% CI: 1.3–5.1; *P*=0.04 in log-rank test) (Figure 5A). There was no significant differences in overall survival in the corresponding D+I subgroups; when the Pr(Decr-pPDGFR) >0.5, median survival was 21 months (95% CI: 19.2 to 30+) and when Pr(Decr-pPDGFR) was ⩽0.5, overall survival was 22 months (95% CI: 12.1 to 30+) (Figure 5B). The HR for the comparison in overall survival between these subgroups in the imatinib-containing arm was 0.96 (95% CI: 0.33–2.78; *P*=0.94 in log-rank test). In the multivariate analysis, a Cox regression model was fit for overall survival (Table 2) including treatment arm assignment, Pr(Decr-pPDGFR) >0.5 with treatment arm, haemoglobin, serum alkaline phosphatase, history of any prior chemotherapy exposure, and baseline ECOG performance score. The proportional hazard assumption was valid (*P*=0.757). The only independent factor predicting survival was Pr(Decr-pPDGFR) >0.5 in docetaxel plus placebo arm (HR=3.24, *P*=0.049).

## Discussion

Preclinical evidence suggested that PDGF signalling contributes to the progression of bone metastases from prostate cancer. Although PDGFR inhibitor therapy enhanced taxane efficacy in orthotopic models of bone metastases (Uehara *et al*, 2003; Kim *et al*, 2006), this was not confirmed in translation to the clinic with a randomised trial of docetaxel chemotherapy with and without the PDGFR inhibitor, imatinib mesylate (Mathew *et al*, 2007). In the study reported here, we assessed pharmacodynamic measures of inhibition of the target receptor, pPDGFR, with survival outcomes within the control and imatinib-containing arms of the randomised trial. We unexpectedly found a strong association between the probability of decrease in phosphorylation of PDGFR in peripheral blood leukocytes and rising plasma PDGF isoform concentrations with inferior PFS and overall survival following docetaxel plus placebo therapy in men with castration-resistant prostate cancer. As these plasma PDGF kinetic profiles were absent and the association of probability of decrease in phosphorylation in peripheral blood leukocytes with PFS and overall survival significantly weaker in the imatinib-containing arm, a confounding effect of imatinib on these associations via PDGF-dependent or PDGF-independent mechanisms is surmised. Interestingly however, on multivariate analysis, Pr(Decr-pPDGFR) was significant also in predicting PFS in the imatinib-containing arm; this observation is reinforced by the hierarchical PFS outcomes by Pr(Decr-pPDGFR) in Figure 4C, suggesting that imatinib effects may narrow but not eliminate completely the predictive differences noted on the docetaxel-alone arm.

Data from this study suggest that a rise in circulating PDGF dimers, associated with a decreased probability of PDGFR phosphorylation in peripheral blood leukocytes relative to baseline following docetaxel therapy, may mark and/or explain resistance to therapy. Further, these data suggest the potential to identify, using these biomarkers, biological subgroups, host-defined and/or tumour-defined, marked by different PFS and overall survival outcomes following docetaxel therapy. As decreased probability of phosphorylated PDGFR in peripheral blood leukocytes predicts inferior outcomes after docetaxel therapy, it may be restated simply that an increased probability of phosphorylated PDGFR in peripheral blood leukocytes predicts improved PFS and overall survival outcomes.

The limitations of this study must be emphasised as these are retrospective analyses of the predictive value of these candidate biomarkers of therapeutic outcome. Specifically, the unexpected association of PDGFR phosphorylation in peripheral blood leukocytes with taxane efficacy requires prospective validation and these studies are planned in the phase III setting where larger sampling is feasible; for example, a sample size of 180 is required to confirm a HR of 2.3 for PFS with Pr(Decr-pPDGFR) >0.5 with a power of 0.82. Whether these findings will prove relevant to the particular dose and schedule of docetaxel studied, to the particular disease state in prostate cancer or to other taxane-responsive neoplasms, will also require prospective verification. Given the findings from multivariate analysis suggesting a persistent, although weaker, predictive effect of Pr(Decr-pPDGFR) for PFS in the imatinib-containing arm (Table 2), the effects of other novel therapeutics with a pPDGFR inhibitory spectrum such as sunitinib or dasatinib in combination with docetaxel, upon the predictive effect of Pr(Decr-pPDGFR) is also of particular interest in prospective validation studies. Validated biomarkers that predict efficacy of docetaxel therapy in metastatic castration-resistant prostate cancer may be useful in partitioning subgroups of patients that could benefit from early rotation to alternative therapeutics when a poor anticipated outcome (PFS of 3 months when Pr(Decr-pPDGFR) >0.5) is predicted with continued docetaxel therapy. Additional work is necessary to assess whether the activation state of other signalling molecules, downstream or parallel to PDGFR activation are superior predictors of docetaxel efficacy.

Further implications of these data are that a mechanistic explanation for a link between dynamic changes in plasma PDGF and phosphorylated PDGFR in peripheral blood leukocytes with docetaxel efficacy would be valuable. Increasing peripheral blood leukocyte PDGFR phosphorylation may mirror effective antivascular and immunological mechanisms in the stroma or the direct antitumor effects of docetaxel targeting microtubules. Taxanes have been hypothesised to function as lipopolysaccharide-mimics capable of inducing a variety of genes in macrophages such as tumour necrosis factor-*α*, interferon-*γ*, granulocyte macrophage-colony stimulating factor, interleukin-1*β* and interleukin-12 which may enhance host immune response, modulate angiogenesis or function as direct tumoricidal agents (Chan and Yang, 2000; Fitzpatrick and Wheeler, 2003). Although formal experimental evidence of *in vivo* activation of PDGFR in peripheral blood leukocytes by taxanes is awaited, it is plausible that PDGFR activation among these cell populations is a marker of a taxane-responsive immunophenotype. As rises in plasma PDGF isoforms are identified in subgroups with an inferior progression-free interval, this may reflect release into circulation by resistant tumour and accompanying neoplastic vasculature and/or decreased binding by immunomodulatory cells lacking PDGFR activation. The pre-operative model in progressive castration-resistant non-metastatic prostate cancer provides an experimental platform to study such questions and narrow such hypotheses. In this model, an association between peripheral blood leukocyte PDGFR phosphorylation and plasma PDGF kinetics following pre-operative docetaxel therapy with spatial and quantitative assessment of tissue PDGF and phosphorylated PDGFR activation in the neoplastic, vascular and stromal components of tumour microenvironment at surgery will be studied. Correlation of these findings with pre-operative tumour regression, PFS and overall survival will provide refinements in mechanistic hypotheses linking docetaxel efficacy and PDGFR phosphorylation.

In summary, the data from this study show that pharmacodynamic monitoring of target inhibition and correlation with therapy outcomes continue to be relevant considerations in clinical trial design. In this respect, the advantages of randomised studies are emphasised by particular observations in the control arm that have led to a new direction of investigation pertaining to docetaxel efficacy. A particular contrast with this predictive strategy is worth drawing with studies of post-therapy declines in PSA or circulating tumour cells, which similar to estrogen-receptor, HER2, and Oncotype DX in breast cancer, are tumour-derived predictors of therapeutic outcomes. Post-therapy pPDGFR dynamics in peripheral blood leukocytes by contrast may reflect pharmacogenomic determinants of taxane metabolism present in both normal somatic cells as well as host-derived tumour cells.

## Figures and Tables

**Table 1:** Plasma platelet-derived growth factor (PDGF) isoform concentrations (ng ml^−1^) at baseline by treatment group: mean and standard deviation (s.d.)

	**Docetaxel+placebo**			**Docetaxel+imatinib**			***P*-value**
**PDGF ligands**	***N***	**Mean**	**s.d.**	***N***	**Mean**	**s.d.**	**(Wilcoxon signed-rank test)**
AA	52	1.671	0.657	50	1.535	0.738	0.102
BB	52	0.569	0.313	50	0.496	0.346	0.267
AB	52	3.25	2.185	50	3.04	2.975	0.338

**Table 2:** Multivariate analysis of progression-free and overall survival

	**Estimate**	**s.d.**	**HR**	***P*-value**
*Progression-free survival*				
Imatinib therapy	0.272	0.299	1.312	0.365
Pr(Decr-pPDGFR)>0.5: Imatinib	0.692	0.331	1.998	0.037
Pr(Decr-pPDGFR)>0.5: Placebo	0.884	0.363	2.419	0.015
log(PDGF at C2D1)−log(PDGF at BL)	−0.325	0.300	0.723	0.122
Hemoglobin ⩾11 g dl^−1^	−1.262	0.444	0.283	0.0045
Elevated alkaline phosphatase	0.461	0.247	1.59	0.061
Prior chemotherapy	0.018	0.123	1.018	0.885
ECOG performance score=2	−0.125	0.521	0.883	0.811
				
*Overall survival*				
Imatinib therapy	0.856	0.626	2.353	0.172
Pr(Decr-pPDGFR)>0.5: Imatinib	0.249	0.612	1.283	0.684
Pr(Decr-pPDGFR)>0.5: Placebo	1.192	0.606	3.293	0.049
log(PDGF at C2D1)−log(PDGF at BL)	−0.043	0.399	0.957	0.913
Hemoglobin ⩾11 g dl^−1^	−0.669	0.717	0.512	0.351
Elevated alkaline phosphatase	0.829	0.439	2.291	0.059
Prior chemotherapy	0.297	0.204	1.346	0.145
ECOG performance score=2	0.625	0.739	1.868	0.398

BL=baseline; C2D1=cycle 2 day 1; ECOG=Eastern Cooperative Oncology Group; HR=hazard ratio; PDGF=total plasma PDGF (AA+BB+AB); Pr(Decr-pPDGFR)=probability of decrease in phosphorylated platelet-derived growth factor receptor; s.d.=standard deviation.
